# Purification and biochemical characterization of FrsA protein from *Vibrio vulnificus* as an esterase

**DOI:** 10.1371/journal.pone.0215084

**Published:** 2019-04-05

**Authors:** Xiaoqin Wang, Zhi-Min Li, Qingyue Li, Mingsong Shi, Lingling Bao, Dingguo Xu, Zhimin Li

**Affiliations:** 1 College of Bioscience and Bioengineering, Jiangxi Key Laboratory for Conservation and Utilization of Fungal Resources, Jiangxi Agricultural University, Nanchang, Jiangxi, China; 2 College of Science, Jiangxi Agricultural University, Nanchang, Jiangxi, China; 3 College of Chemistry, Sichuan University, Chengdu, Sichuan, China; 4 Collaborative Innovation Center of Postharvest Key Technology and Quality Safety of Fruits and Vegetables in Jiangxi Province, Nanchang, Jiangxi, China; Shantou University Medical College, CHINA

## Abstract

Fermentation-respiration switch protein (FrsA) was thought to play an important role in controlling the metabolic flux between respiration and fermentation pathways, whereas the biochemical function of FrsA was unclear yet. A gene coding for FrsA protein from *Vibrio vulnificus* was chemically synthesized. The recombinant VvFrsA was expressed as a soluble protein and purified by Ni-NTA affinity chromatography. The protein had a subunit molecular weight of *ca*. 45 kDa by SDS-PAGE and preferred short-chain esters when p-nitrophenyl alkanoate esters were used as substrates. Optimum condition for VvFrsA was found to be at pH 9.0 and 50 °C. The protein retained high esterase activity at alkaline condition and would denature slowly at over 50 °C. With p-nitrophenyl acetate as the substrate, the *K*_m_ and *k*_cat_ were determined to be 18.6 mM and 0.67 s^-1^, respectively, by steady-state kinetic assay. Molecular dynamics simulation and docking model structure revealed that p-nitrophenyl acetate could be the substrate of VvFrsA. In conclusion our results demonstrated that the protein was able to catalyze the hydrolysis of esters, especially p-nitrophenyl acetate, for the first time.

## Introduction

Fermentation-respiration switch protein (FrsA) was initially identified as a novel protein binding to the unphosphorylated form of IIA^Glc^ specifically in *Escherichia coli* extracts [1]. FrsA was thought to be involved in the metabolic flux between respiration and fermentation pathways by interacting with IIA^Glc^ in *E*. *coli* [1]. Disruption of *frsA* gene in *E*. *coli* resulted in increased respiration rate when glucose was used as carbon source, whereas overexpression of FrsA accelerated fermentation rate on glucose with concurrent accumulation of mixed-acid fermentation products [1]. Although the physiological function of FrsA was implicated in metabolism, its biochemical function was not clear yet.

It was proposed that pyruvate might be the biochemical substrate of FrsA since pyruvate catabolism was the branching point between respiration and fermentation in glucose metabolism pathway [2]. To test this hypothesis, recombinant FrsA from *V*. *vulnificus* (hereafter VvFrsA) was selected and purified within *E*. *coli* overexpression system due to its higher expression in this system compared to other orthologs, which existed in various facultative anaerobes [1]. As a result, Lee et al. claimed that VvFrsA functioned as a cofactor-independent pyruvate decarboxylase with highest activity reported so far [2]. In contrast, Kellett et al. pointed out that VvFrsA was not a cofactor-independent pyruvate decarboxylase with computational, structural and kinetic evidences afterwards [3]. In fact, only orotidine-5’-monophosphate decarboxylase and 2-oxo-4-hydroxy-4-carboxy-5-ureidoimidazoline decarboxylase were described as cofactor-independent decarboxylases so far [4, 5]. In these two enzymes, the decarboxylation force was believed to be the electrostatic repulsion between the two carboxylate groups located in the substrate and enzyme, respectively [4, 5]. Usually, cofactor (for instance, TPP) was required for α-keto acid decarboxylase such as pyruvate decarboxylase, in which the cofactor functioned as an electron sink to stabilize the carbanion developed upon decarboxylation [6].

Crystal structures of VvFrsA indicated that it was one member of α/β hydrolase superfamily [2, 3]. The enzymes in this superfamily share similar topologies consisting of eight parallel β strands and six α helices usually [7]. Various catalytic functions such as hydrolase, transferase, oxidoreductase and C-C bond-cleaving functions have evolved from this α/β hydrolase fold [8]. As a result, members of this hydrolase superfamily displayed broad applications in food and pharmaceutical industries [9]. Esterases (EC 3.1.1.1) catalyze the hydrolysis of ester substrates with short to medium acyl chains via the Ser-Asp/Glu-His catalytic triad [10, 11]. VvFrsA was designated as an esterase based on amino acid sequence homology in NCBI database. Therefore, it was speculated that VvFrsA might catalyze hydrolysis of certain esters in vitro.

In this study, the gene encoding VvFrsA with six histidines in the C-terminus was chemically synthesized. The recombinant VvFrsA was purified to be homogeneous and the substrate specificity of VvFrsA was assayed. Experimental results showed that VvFrsA preferred short chain alkanoate rather than long chain alkanoate as substrate. Furthermore, the kinetic and biochemical characteristics of VvFrsA towards pNPA were determined in detail for the first time.

## Materials and methods

### Reagents and chemicals

Ni-NTA agarose resin was purchased from Qiagen (Hilden, Germany). *Trans*5K DNA marker, T4 DNA ligase, *E*. *coli Trans*5α and BL21(DE3) competent cells were products of TransGen Biotech (Beijing, China). *Nde*I and *Xho*I endonucleases were purchased from New England Biolabs (Ipswich, MA, USA). The substrates of pNPA, pNPB, pNPC, pNPL and pNPP were obtained from Aladdin Biotech (Shanghai, China).

### Construction of pET28a-VvFrsA plasmid

Gene encoding VvFrsA with *Nde*I and *Xho*I restriction sites at 5’ and 3’-end, respectively, was synthesized according to the codon preference in *E*. *coli* by Invitrogen (Shanghai, China). *Nde*I and *Xho*I digested *VvFrsA* gene was ligated with pET-28a vector cleaved with the same endonucleases by T4 ligase at 16 °C overnight. Then the constructed vector of pET28a-VvFrsA was transformed into *E*. *coli Trans*5α competent cell and the cell was cultured on Luria broth (LB) agar plate containing kanamycin (50 μg/mL) at 37 °C overnight. Plasmid of pET28a-VvFrsA was prepared from a single clone and then verified by DNA sequencing.

### Expression and purification of recombinant VvFrsA protein

Plasmid of pET28a-VvFrsA was transformed into *E*. *coli* BL21(DE3) competent cells and then the cells were plated on solid LB media containing kanamycin (50 μg/mL). Positive single clone was picked up and cultivated in liquid LB media containing kanamycin (50 μg/mL) at 37 °C, 180 rpm overnight. The overnight cell culture was added to fresh LB media containing kanamycin (50 μg/ml) with the ratio of 1:100 and the media was shaken at 37 °C, 180 rpm until OD_600nm_ ~0.6. Then the cell culture was cooled to 20 °C and isopropyl-β-D-thiogalactopyranoside (IPTG, 0.2 mM) was added to trigger the expression of VvFrsA at 20 °C, 180 rpm for 24 h. The cells were harvested by centrifuging at 4 °C, 6,500 rpm for 15 min.

Purification of recombinant VvFrsA was achieved according to the described procedures [12]. Specifically, cell pellets were resuspended in lysis buffer (20 mM Tris-HCl, pH 7.5) at 4 °C and cell suspension was sonicated in ice-water bath. Then the cell lysate was centrifuged at 4 °C, 12,000 rpm for 2 h. The supernatant after being filtrated with 0.45 μm membrane was loaded to Ni-NTA column, which was previously equilibrated with lysis buffer at 4 °C. The column was washed with linear gradient 0–200 mM imidazole in lysis buffer. Elution fractions were monitored by measuring their absorbance at 280 nm and analyzed by SDS-PAGE. The fractions containing VvFrsA protein were pooled, concentrated and dialyzed over a lysis buffer containing 300 mM NaCl, 1 mM dithiothreitol (DTT). The concentration of VvFrsA was determined by measuring the protein absorbance at 280 nm with molar extinction coefficient of 74,955 M^-1^ cm^-1^.

### Substrate specificity of VvFrsA towards p-nitrophenyl alkanoate esters

The substrate specificity of VvFrsA towards pNPA, pNPB, pNPC, pNPL and pNPP was analyzed based on the protocol proposed by Erdmann et al. with slight modification [13]. Solution 1 was prepared by dissolving the aforementioned substrates in isopropanol respectively. Solution 2 consisted of 50 mM phosphate buffer at pH 7.5 containing 0.4% Triton X-100. Before the reaction, solution 2 was kept at a constant temperature by placing it in a 37 °C water bath. The reaction mixtures include one specific substrate with final concentration of 0.1 mM in a mixed solution of solution 1 and solution 2 with a ratio of 1:9. The reaction was started by adding VvFrsA with final concentration of 20 μM to the reaction mixtures at 37 °C. The absorbance of the colored reaction solution was recorded continuously for 10 min at 410 nm using spectrophotometer. The reaction rate was calculated according to the Lambert-Beer’s Law with molar extinction coefficient of p-nitrophenol as 15,000 M^-1^ cm^-1^.

### Effects of pH and temperature on activity of VvFrsA towards pNPA

The catalytic hydrolysis activity of VvFrsA at pH range of 6.5–10.0 was determined by the aforementioned continuous spectrophotometric method. The following buffers were applied in the assay: 50 mM Bis-Tris for pH 6.5; 50 mM PBS for pH 7.0; 50 mM Tris-HCl for pH 7.5, 8.0 and 8.5; 50 mM CHES for pH 9.0 and 9.5; 50 mM CAPS for pH 10.0. The standard reaction mixture includes 1 mM pNPA and 3.8 μM VvFrsA in the above respective buffers. The effects of temperature within the range of 0–80 °C on the enzymatic activity of VvFrsA were assayed via similar method. The reaction was repeated three times. All the background hydrolysis rates were subtracted in calculating the activity of VvFrsA.

### Kinetic constants of VvFrsA towards pNPA

The *K*_m_ and *k*_cat_ constants of VvFrsA towards pNPA were determined with the aforementioned continuous spectrophotometric assay. Specifically, the reaction mixtures (500 μL) contained 9.5 μM VvFrsA and various concentrations of pNPA (0.05–6.0 mM) in buffer of 50 mM PBS (pH7.5). The reaction rate without VvFrsA was used as control. The initial reaction rates at different substrate concentrations were fitted to the Michaelis-Menten equation by KaleidaGraph software. All reactions were performed in triplicate.

### Theoretical models

The initial structure of VvFrsA was extracted from the crystal structure (PDB code: 4I4C) [3]. The pNPA structure was downloaded from the ZINC database [14] and optimized using B3LYP/6-31G [15, 16]. The missing hydrogen atoms of VvFrsA protein were added using leap module in Amber12 program. The partial charges for both VvFrsA and pNPA were assigned using ADT with Gasteiger method [17]. A grid map of 60×60×60 points with 0.375 Å grid spacing was generated using AutoGrid module [18]. For docking with pNPA, the original center of n-hexanoic acid in crystal structure of VvFrsA was used as the center for making the map. For searching the docking conformation, the Lamarckian genetic algorithm was used as the searching approach [19]. A total of 2000 automated docking runs were finally generated to find all the conformations of pNPA in the active site of VvFrsA. Then, the docked conformation with the lowest energy was selected as the initial model for further MD simulation.

Gaussian09 software was used to optimize the geometry of pNPA at HF/6-31G level of theory. Subsequently, we used the restrained electrostatic potential (RESP) protocol to calculate the partial atomic charges and electrostatic potential calculations using B3LYP/6-31G* method [15, 16, 20]. At the end, the force field parameters for the ligands were obtained by using the Antechamber program [21]. The standard AMBER ff14SB force field was used to describe VvFrsA protein [22]. The obtained systems were solvated by a pre-equilibrated rectangular box of TIP3P water [23]. The whole system was neutralized with chloridion. We used three steps to minimize the complex systems to remove the bad contacts in the solvated complex systems. In the first place, the positions of water molecules were minimized by 10000 steps of steepest descent. Next, the positions of non-backbone atoms of proteins were minimized by 10000 steps of steepest descent. Thirdly, further 10000 steps of conjugate gradient approach were carried out for total system. The non-bond interactions were dealt with 12 Å cutoff and the periodic boundary conditions were applied. The long-range electrostatic interactions were described by the particle mesh ewald algorithm [24]. In the MD simulation, Newton’s equations of atomic motion were integrated with 2 fs time step by the Verlet algorithm. SHAKE algorithm was used to constrain the covalent bonds involving hydrogen atoms [25]. All calculations were performed using the AMBER12 suite of programs.

## Results

### Construction of pET28a-VvFrsA plasmid

**Fig 1:**
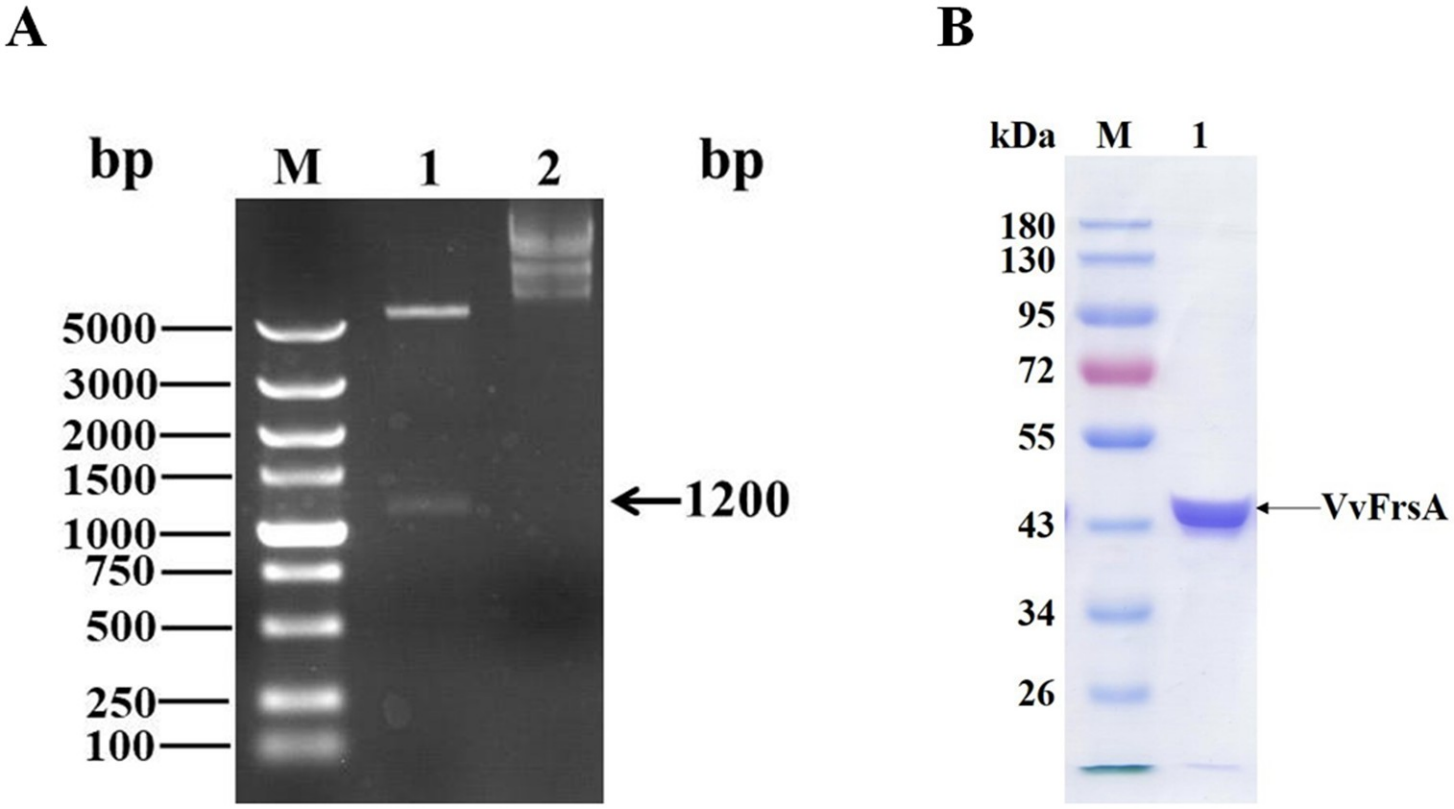
Construction of expression plasmid and purification of VvFrsA. **A**. Agarose gel of pET28a-VvFrsA plasmid. M: DNA marker; 1: pET28a-VvFrsA plasmid after digestion with *Nde*I and *Xho*I; 2: plasmid of pET28a-VvFrsA. **B**. SDS-PAGE gel of purified VvFrsA. M: protein marker; 1: purified VvFrsA.

The gene encoding VvFrsA with six histidines in the C-terminus was chemically synthesized according to the codon preference in *E coli*. (S1 Fig). The recombinant plasmid was double digested with *Nde*I and *Xho*I endonucleases, and two bands appeared after agarose gel electrophoresis. As shown in Fig 1A, the upper fragment over 5000 bp was pET-28a vector, and the lower fragment was approximately 1.2 kb, which was consistent with the theoretical number of base pairs of *VvFrsA* gene (1248 bp) (band 1 in Fig 1A). Furthermore, the deduced amino acid sequence of synthesized *VvFrsA* gene was exactly same as the amino acid sequence of VvFrsA protein (protein ID: WP_011078432). Therefore recombinant plasmid of pET28a-VvFrsA was successfully constructed.

### Purification and characterization of VvFrsA

Recombinant VvFrsA protein was expressed and purified previously [2, 3]. Different from previous report, in which pQE vector was used to construct the expression plasmid [2, 3], pET28a vector was used in this study. Therefore the plasmid of pET28a-VvFrsA was transformed into *E*. *coli* BL21(DE3) competent cell and recombinant VvFrsA was purified to be homogeneous with a yield of 6 mg/g wet cells by using Ni-NTA affinity chromatography. The subunit molecular mass of VvFrsA was estimated to be *ca*. 45 kDa and the purity was estimated to be over 95% by SDS-PAGE (Fig 1B). The native mass of VvFrsA was determined to be 98 kDa by size exclusion chromatography, which demonstrated that VvFrsA was a dimer considering the theoretical mass of VvFrsA was *ca*. 47 kDa. This result was in line with crystal structure data of VvFrsA that two chains existed in one unit cell [3].

### Substrate specificity of VvFrsA towards p-nitrophenyl alkanoate esters

**Fig 2:**
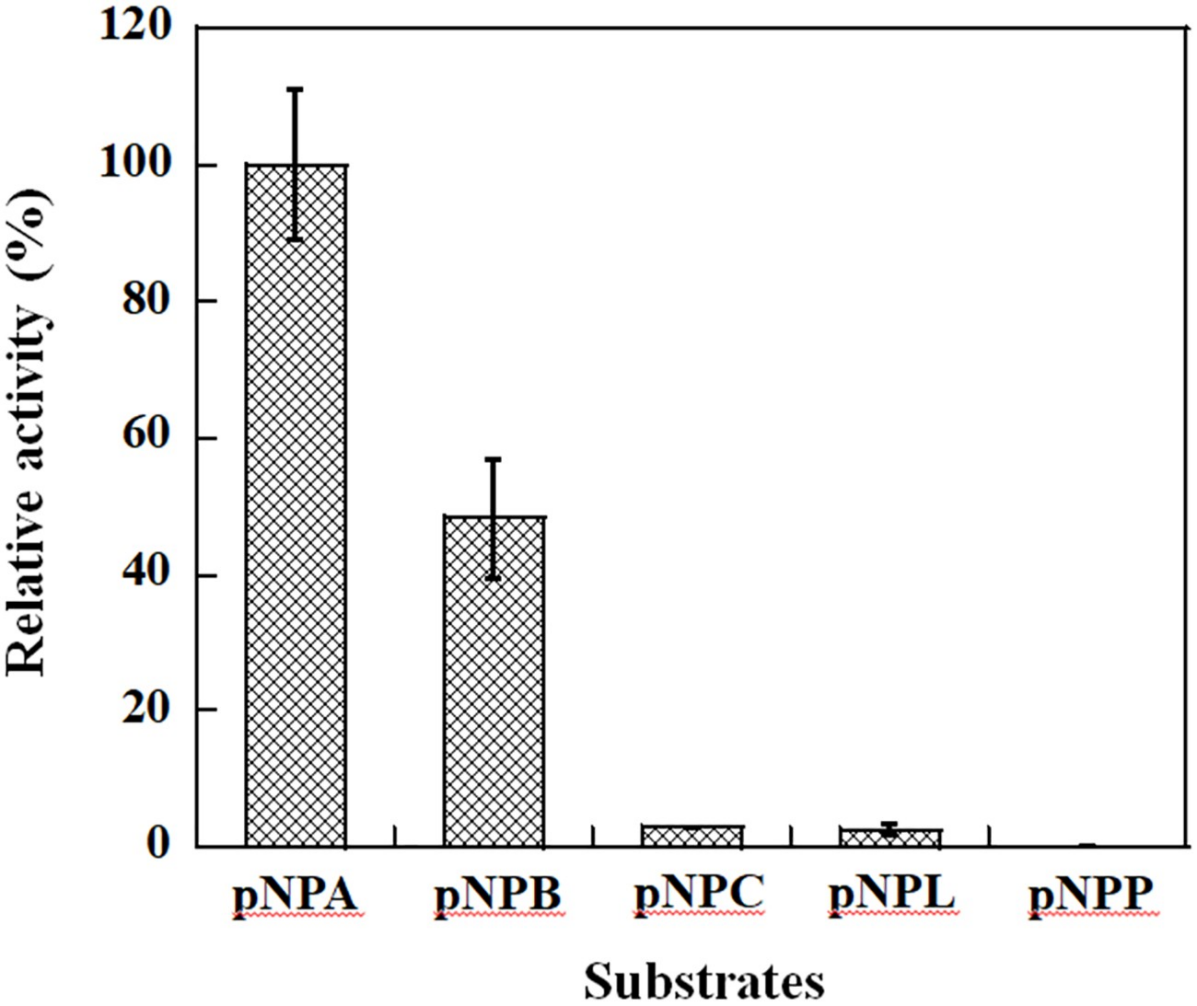
Relative activity of VvFrsA towards p-nitrophenyl alkanoate esters. Hydrolysis of p-nitrophenyl esters was expressed as a percentage compared with pNPA hydrolysis. There was no activity with pNPP substrate. Experiments were repeated three times.

Crystal structures of VvFrsA demonstrated that it was one member of α/β hydrolase superfamily [2, 3], which catalyzed a wide range of reactions including hydrolysis of esters [26]. As a result, p-nitrophenyl alkanoate esters were used as model substrates to evaluate the hydrolytic activity and substrate specificity of VvFrsA. Among the tested substrates, VvFrsA displayed highest activity towards pNPA as shown in Fig 2. The reactivity of VvFrsA towards pNPB was only about 50% of that towards pNPA (Fig 2). When the length of acyl increased to eight or twelve carbons, the reactivity of VvFrsA decreased significantly to less than 5% of that of pNPA (Fig 2). VvFrsA couldn’t catalyze the hydrolysis of pNPP to produce p-nitrophenol (Fig 2). Therefore it was reasonable to conclude that VvFrsA preferred short chain alkanoate ester rather than long chain counterparts when p-nitrophenyl alkanoate esters were used as substrates. These results indicated that VvFrsA was an esterase and not a lipase (EC 3.1.1.3), which usually catalyzes the hydrolysis of esters with long chains of more than 10 carbons [27].

### Effects of pH and temperature on activity of VvFrsA towards pNPA

**Fig 3:**
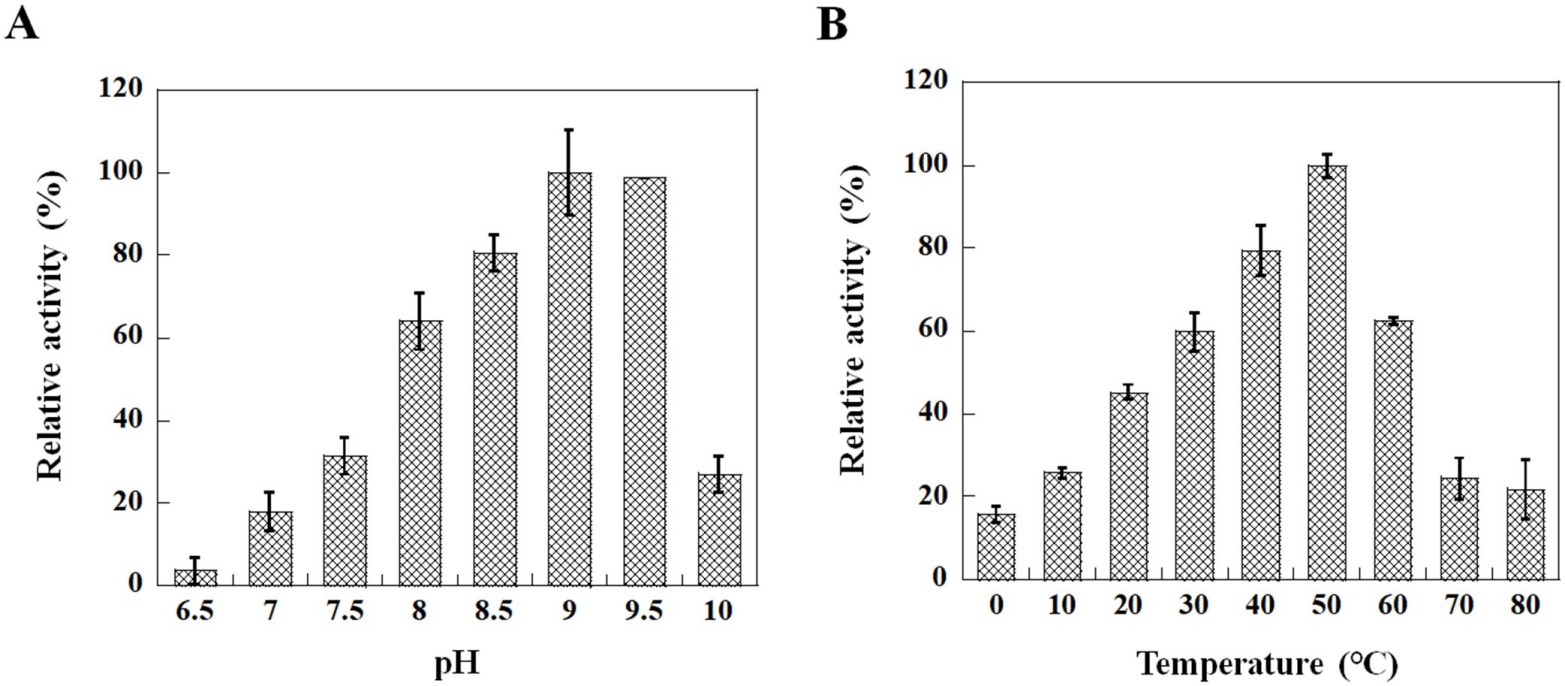
Effects of pH and temperature on hydrolytic activity of VvFrsA. **A**. pH dependence of VvFrsA. The assays were carried out with 1 mM pNPA as substrate and 3.8 μM VvFrsA in various buffer at room temperature. **B**. Temperature effect on activity of VvFrsA. The assays were carried out with 1 mM pNPA as substrate and 2.0 μM VvFrsA in the buffer of 50 mM CHES, pH 9.0. The reaction was quenched after 10 min. Experiments were repeated three times.

The effects of pH and temperature on the hydrolytic activity of VvFrsA were determined spectrophotometrically using pNPA as substrate. As shown in Fig 3A, the optimal pH for VvFrsA was found to be at the range of 9.0–9.5 and the enzyme displayed high activity in alkaline conditions from pH 8–9.5. The activity of VvFrsA would decline significantly when the buffer pH was at 7.5 or 10 (Fig 3A), at which the enzymatic activity was only about one third of that at the optimal pH. This optimal condition at alkaline pH of VvFrsA was similar to other esterases from halophilic/alkaline environments [28–30], where *V*. *vulnificus* inhabits.

VvFrsA displayed increasing activity in the temperature range of 0–50 °C with highest activity at 50 °C (Fig 3B). The enzyme retained 60% of its highest activity at 60 °C, and the activity at 70 °C was only 25% of its highest activity (Fig 3B). These results indicated that VvFrsA was moderately thermostable. Meanwhile, the activity of VvFrsA would not change significantly during 30 min incubation at temperature below 50 °C. This observation was in agreement with the effect of temperature on activity of esterase EstOF4 from *Bacillus pseudofirmus* [28].

### Steady-state kinetic constants of VvFrsA

**Fig 4:**
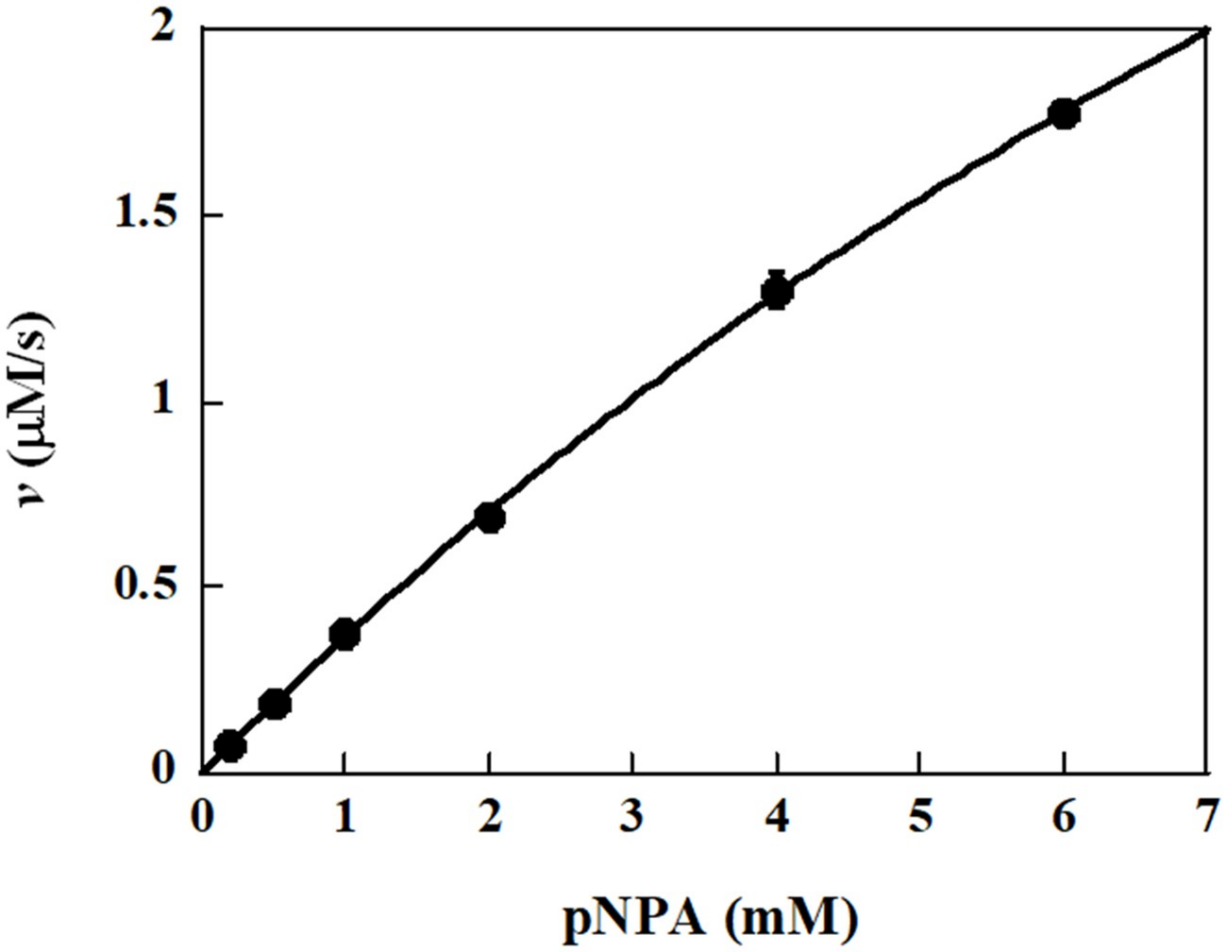
Kinetic analysis of VvFrsA with pNPA as substrate. The initial velocities were determined as function of pNPA substrate concentrations with the concentration of VvFrsA fixed at 9.5 μM in the buffer of 50 mM PBS, pH 7.5.

Since the spontaneous hydrolysis of pNPA at pH 9.0 buffer was fast, the steady-state kinetic constants of VvFrsA were determined in 50 mM PBS, pH 7.5 at 25 °C. The initial reaction velocity of VvFrsA dependent on concentration of pNPA was illustrated as Fig 4. By fitting the initial reaction velocities and substrate concentrations to the Michaelis-Menten equation, *K*_m_ of pNPA and maximum initial velocity were determined to be 18.6 ± 1.3 mM and 6.4 ± 0.4 μM/s, respectively. Therefore, the *k*_cat_ and *k*_cat_/*K*_m_ were calculated to be 0.67 s^-1^ and 36 M^-1^ s^-1^, respectively, considering the enzyme concentration was 9.5 μM.

## Discussion

VvFrsA was biochemically identified to function as a cofactor-independent pyruvate decarboxylase with the highest activity reported so far [2]. The *k*_cat_ of VvFrsA as a pyruvate decarboxylase was reported to be approximately 1400 s^-1^, which was considerably higher than that of TPP-dependent pyruvate decarboxylase from other species such as *Saccharomyces cerevisiae* [31]. This extraordinary finding, if correct, cast contradiction with current cognition that nature evolved cofactor such as TPP to stabilize the generated acyl carbanions during the oxidative and non-oxidative decarboxylation of α-keto acids in all kingdoms of life. However, this remarkable claim was disputed by Kellett et al., who demonstrated that VvFrsA was not a cofactor-independent pyruvate decarboxylase with computational, structural and kinetic evidences later [3]. With these contradictory observations in mind, we expressed the recombinant VvFrsA with *E*. *coli* expression system and purified the protein by Ni-NTA affinity chromatography.

VvFrsA was predicted to be an esterase based on amino acid sequence homology in NCBI database. In addition, the crystal structure of VvFrsA placed it in α/β hydrolase superfamily, in which esterase was included. Therefore, the esterase activity of VvFrsA was assayed with p-nitrophenyl alkanoate esters as model substrates in this study. Kinetic results revealed that VvFrsA did catalyze the hydrolysis of p-nitrophenyl alkanoate esters to produce p-nitrophenol and pNPA (C2) was the best substrate of VvFrsA among the tested substrates (Fig 2 and S2 Fig). Although esterases have similar structural topology, the substrate specificities are different. For example, the esterase from *Pseudomonas mandelii* preferred to use pNPA (C2) as substrate, whereas esterase from *Bacillus pseudofirmus* preferred the substrate of p-nitrophenyl caproate (C6) [28, 32]. Therefore, the determinants for substrate specificity of VvFrsA need to be elucidated further. At the same time, compared to other esterases, VvFrsA displayed much lower hydrolytic activity towards p-nitrophenyl esters substrates due to the low binding affinity of pNPA with VvFrsA if we simply took *K*_m_ into account (*K*_m_ of pNPA for VvFrsA was 18.6 mM). For instance, with pNPA as substrate, the *K*_m_ and *k*_cat_ were respective 0.21 mM and 3.4 s^-1^ for esterase from *Pseudomonas mandelii* [32], and the *K*_m_ and *k*_cat_ were respective 0.037 mM and 2.07 s^-1^ for esterase from *Bacillus pseudofirmus* [28].

**Fig 5:**
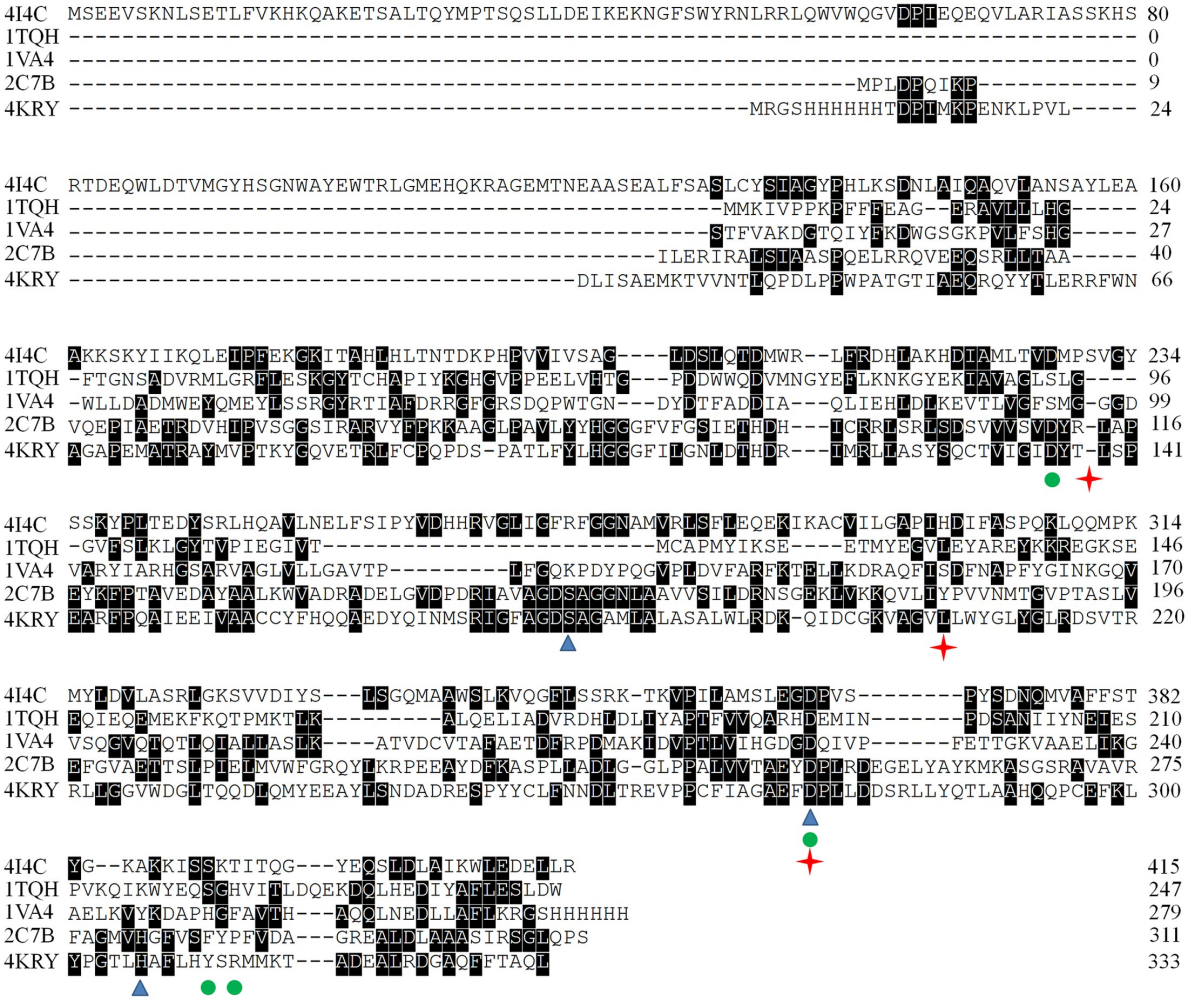
Amino acids sequences alignment of VvFrsA with other esterases from different sources. 4I4C, 1TQH, 1VA4, 2C7B and 4KRY are the representations of esterases from *Vibro vulnificus*, *Geobacillus stearthermophilus*, *Pseudomonas fluorescens*, Metagenomic Library of uncultured archaeon and *Escherichia coli (strain K12)*, respectively. The catalytic triad of 4I4C is indicated by red cross stars, green solid cycles indicate the catalytic triad of 1TQH and 1VA4, and blue triangles indicate the catalytic triad of 2C7B and 4KRY. Sequences alignment was carried out by Clustal W program.

**Fig 6:**
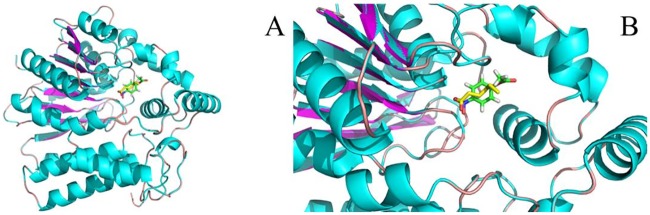
The superimposed structures of the X-ray structure of n-hexanoic acid (PDB ID: 4I4C) and the docked structure of p-nitrophenyl acetate. **A**. The overall illustration. **B**. Magnified active center illustration. Cartoon style used for VvFrsA. The p-nitrophenyl acetate is plotted using stick style which shows C: green, H: gray, N: blue, O: red. The n-hexanoic acid shows with stick style for C: yellow, H: gray, N: blue, O: red.

**Fig 7:**
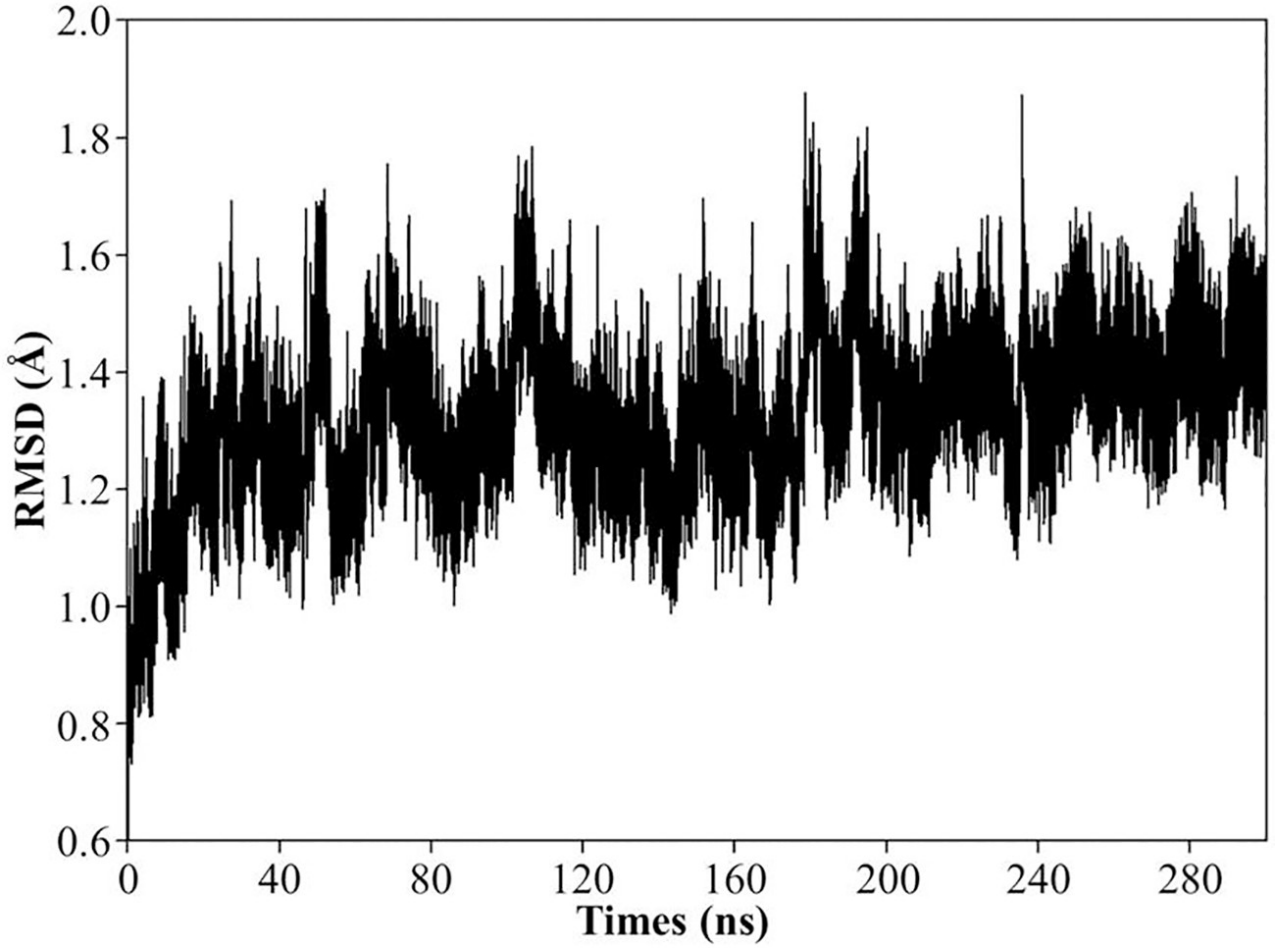
Various RMSD plots from the MD simulations of VvFrsA-pNPA system. Plot of the RMSD as a function of time for the simulations of VvFrsA-pNPA system calculated based on VvFrsA protein backbone atoms in the X-ray structure.

In consideration of the low activity of VvFrsA towards pNPA, pNPA might not be the native substrate of VvFrsA. However, the catalytic triad was conserved in VvFrsA as other esterases despite of their low amino acids sequences identities (Fig 5). The typical amino acid sequence identities of VvFrsA with other esterases were about 10–20%. It is worth pointing out that the conserved catalytic triad was not aligned in various esterases (Fig 5). Nevertheless, we docked pNPA into the crystal structure of VvFrsA (PDB ID: 4I4C) to get the model complex structure of VvFrsA with pNPA (Fig 6). The complex structure revealed that pNPA was well superimposed with the n-hexanoic acid in the active center of VvFrsA. In addition, MD simulations results showed that the complex of pNPA with VvFrsA was stable within 300 ns scale (Fig 7 and S3 Fig). Therefore, it is reasonable to conclude that VvFrsA functions as esterase biochemically. On the other hand, the model complex structure revealed that the catalytic triad was located on the loop structure of VvFrsA and far away the docked pNPA, which might contribute to the low activity of VvFrsA towards pNPA (S4 Fig). Anyway, the model complex structure would facilitate the discovery of native substrate of VvFrsA and elucidate the structure-function relationship of VvFrsA in future.

## Conclusion

In this study, pET28a-VvFrsA expression vector was constructed and recombinant VvFrsA protein was purified by Ni-NTA affinity chromatography. Based on the amino acid sequence homology and the crystal structure of α/β hydrolase fold, the hydrolytic activity of VvFrsA for esters was determined for the first time. Our results demonstrated that the protein was able to catalyze the hydrolysis of esters, especially p-nitrophenyl acetate, and the protein was kinetically characterized with p-nitrophenyl acetate as substrate in detail.

## Supporting information

S1 Fig Nucleotide sequence of synthesized *VvFrsA* gene with eighteen bases coding for six histidines in 3’ end. The synthesized gene sequence was virtually same as the nucleotide sequence with GenBank accession number of NC_005139.
(TIF)

S2 Fig Substrate specificity of VvFrsA towards p-nitrophenyl alkanoate esters. Hydrolysis of p-nitrophenyl esters to give yellow p-nitrophenol. **A**. VvFrsA catalyzed hydrolysis of p-nitrophenyl esters; **B**. Spontaneous hydrolysis of p-nitrophenyl esters without VvFrsA. All substrates concentrations are 1 mM and VvFrsA is 5 μM in 50 mM PBS, pH 7.5.
(TIF)

S3 Fig Molecular dynamics simulation of VvFrsA with pNPA. **A**. Snapshots of VvFrsA with pNPA binding models along the dynamics simulation time. For clarity, the water molecules have been removed. pNPA is plotted using stick style (C: green, H: gray, N: blue, O: red), while cartoon style for VvFrsA (helix: cyan, sheet: magenta, loop: orange). **B**. Various RMSF plots from the MD simulations of VvFrsA-pNPA systems. Plot of the RMSF as a function of residues number for the simulation of VvFrsA-pNPA systems calculated based on VvFrsA protein backbone atoms.
(TIF)

S4 Fig Cartoon structure of model complex of VvFrsA with pNPA. The catalytic triad is located on the loops of VvFrsA (shown as stick style, C: green, H: gray, N: blue, O: red). pNPA is pictured as stick style (C: green, H: gray, N: blue, O: red).
(TIF)

## Abbreviations

Bis-Tris: [bis(2-hydroxyethyl)amino]tris-(hydroxymethyl)methane
CAPS: 3-(*N*-cyclohexylamino)-1-propanesulfonic acid
CHES: 2-(*N*-cyclohexylamino)ethanesulfonic acid
MD: molecular dynamics
NTA: nitrilotriacetic acid
PBS: phosphate buffer saline
pNPA: p-Nitrophenyl acetate
pNPB: p-Nitrophenyl butyrate
pNPC: p-Nitrophenyl caprylate
pNPL: p-Nitrophenyl laurate
pNPP: p-Nitrophenyl palmitate
SDS-PAGE: sodium dodecyl sulphate-polyacrylamide gel electrophoresis
TPP: thiamine pyrophosphate
Tris: tris(hydroxymethyl)aminomethane
