# Supplementary figures and images for: Purification and biochemical characterization of FrsA protein from Vibrio vulnificus as an esterase
Source: PLoS One. 2019 Apr 5;14(4):e0215084. doi: 10.1371/journal.pone.0215084 (PMC6450606; doi:10.1371/journal.pone.0215084)

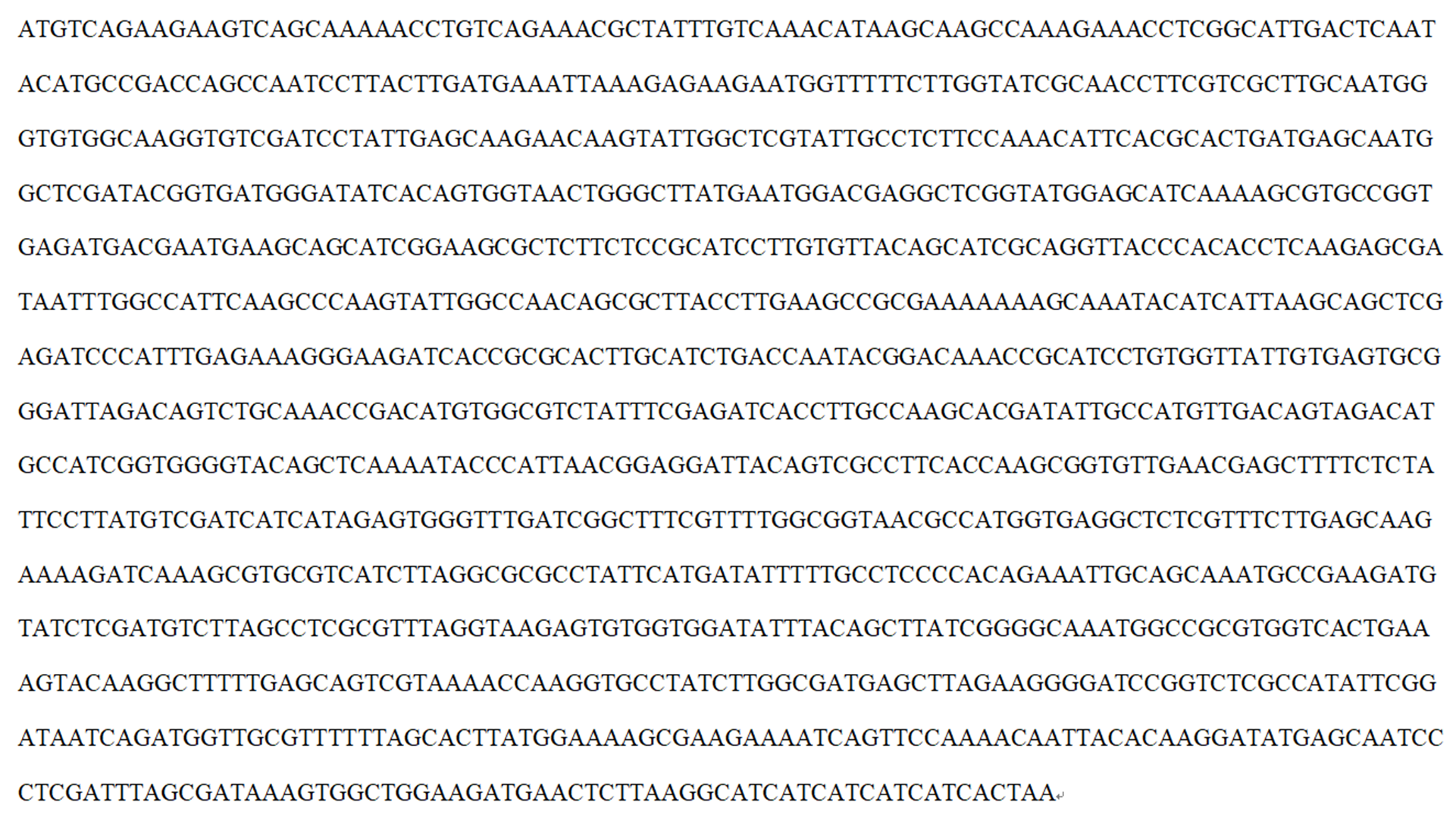

Supplement: S1 Fig — The synthesized gene sequence was virtually same as the nucleotide sequence with GenBank accession number of NC_005139. (TIF) [file pone.0215084.s001.tif]

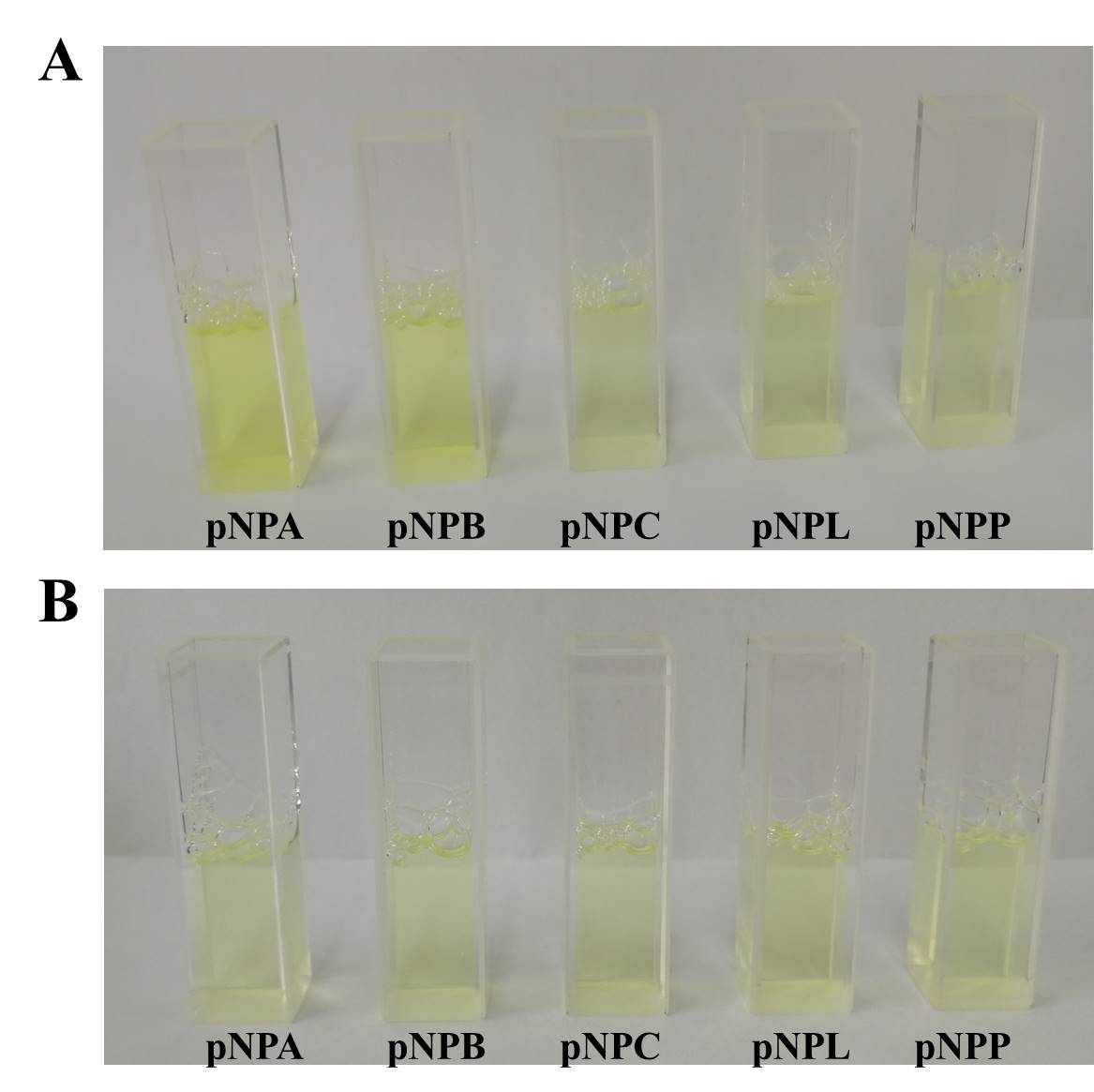

Supplement: S2 Fig — Hydrolysis of p-nitrophenyl esters to give yellow p-nitrophenol. A. VvFrsA catalyzed hydrolysis of p-nitrophenyl esters; B. Spontaneous hydrolysis of p-nitrophenyl esters without VvFrsA. All substrates concentrations are 1 mM and VvFrsA is 5 μM in 50 mM PBS, pH 7.5. (TIF) [file pone.0215084.s002.tif]

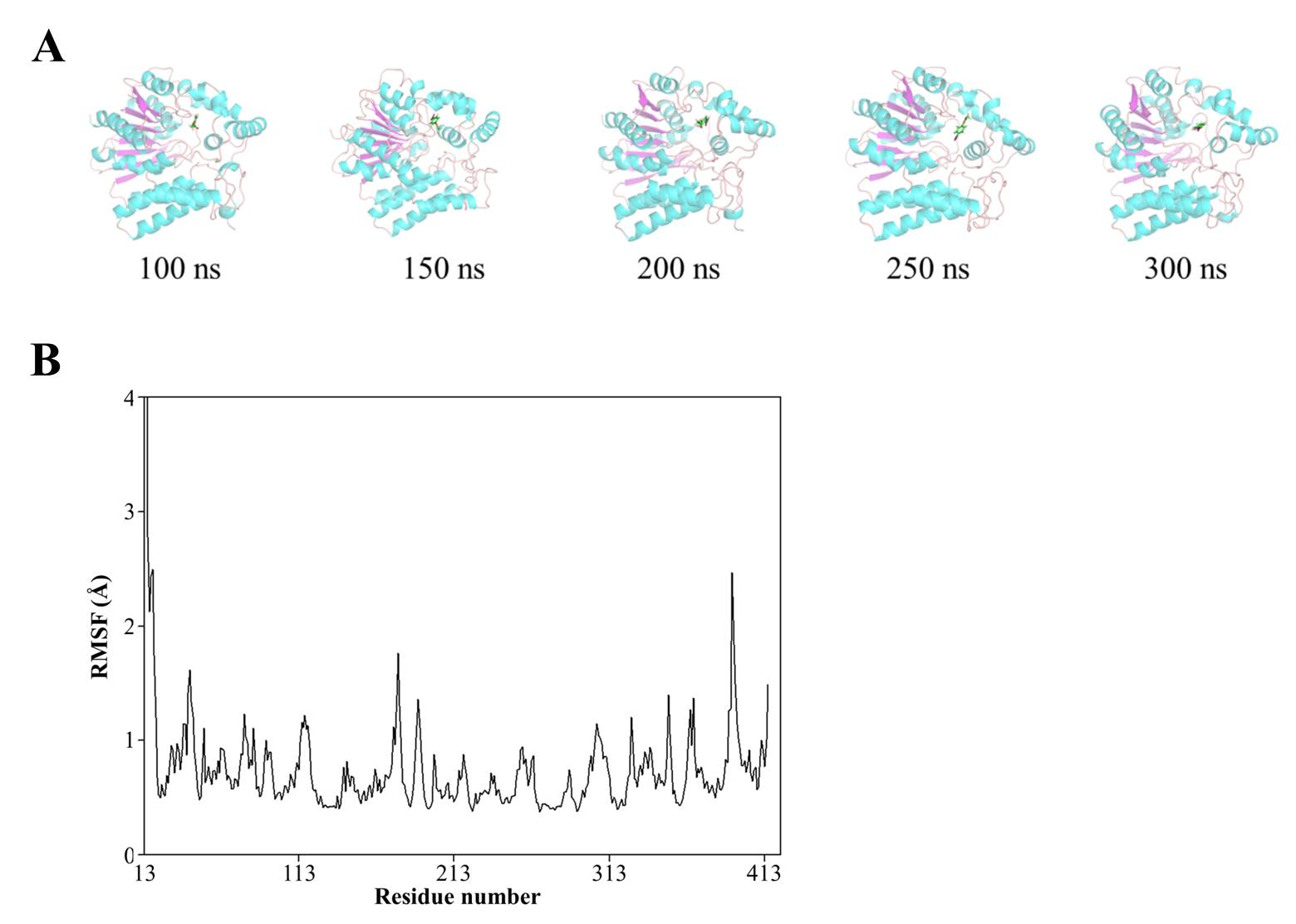

Supplement: S3 Fig — A. Snapshots of VvFrsA with pNPA binding models along the dynamics simulation time. For clarity, the water molecules have been removed. pNPA is plotted using stick style (C: green, H: gray, N: blue, O: red), while cartoon style for VvFrsA (helix: cyan, sheet: magenta, loop: orange). B. Various RMSF plots from the MD simulations of VvFrsA-pNPA systems. Plot of the RMSF as a function of residues number for the simulation of VvFrsA-pNPA systems calculated based on VvFrsA protein backbone atoms. (TIF) [file pone.0215084.s003.tif]

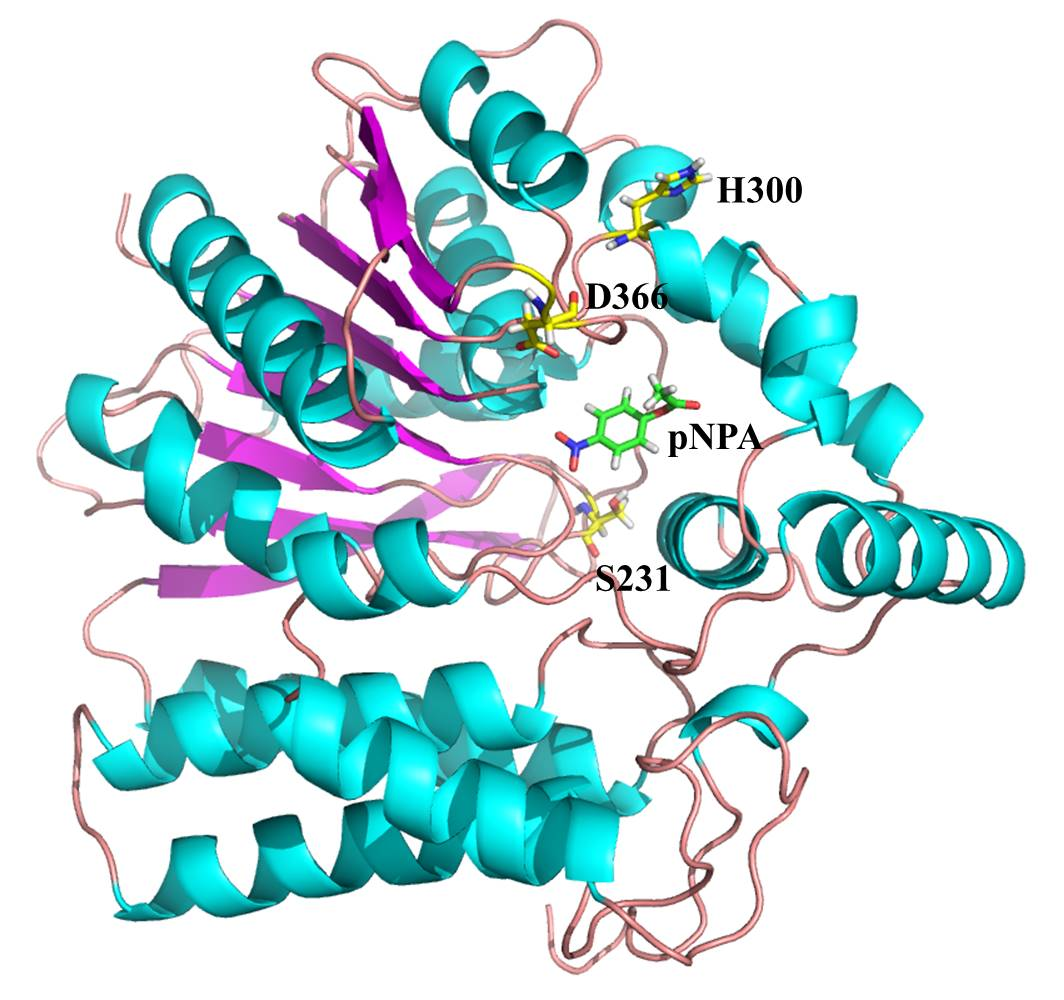

Supplement: S4 Fig — The catalytic triad is located on the loops of VvFrsA (shown as stick style, C: green, H: gray, N: blue, O: red). pNPA is pictured as stick style (C: green, H: gray, N: blue, O: red). (TIF) [file pone.0215084.s004.tif]
